# The *MTDH* (−470G>A) Polymorphism Is Associated with Ovarian Cancer Susceptibility

**DOI:** 10.1371/journal.pone.0051561

**Published:** 2012-12-11

**Authors:** Cunzhong Yuan, Xiao Li, Shi Yan, Qifeng Yang, Xiaoyan Liu, Beihua Kong

**Affiliations:** 1 Department of Obstetrics and Gynecology, Qilu Hospital of Shandong University, Ji'nan, Shandong, China; 2 Gynecology Oncology Key Library of Shandong Province, Qilu Hospital of Shandong University, Ji'nan, Shandong, China; 3 Department of Breast Surgery, Qilu Hospital of Shandong University, Ji'nan, Shandong, China; Kinghorn Cancer Centre, Garvan Institute of Medical Research, Australia

## Abstract

*MTDH*(metadherin), an important oncogene that is widely overexpressed in various cancers, is a potential biomarker of tumor malignancy. Variants in *MTDH* have been associated with susceptibility to breast cancer. However, no studies assessing *MTDH* gene polymorphisms and their potential relationship to ovarian cancer susceptibility have been reported. Thus, we investigated the association of *MTDH* (−470G>A) polymorphism with ovarian cancer development in 145 ovarian cancer patients and 254 matched control subjects, using sequence analysis. We found that the *MTDH* (−470G>A) polymorphism was statistically correlated with ovarian cancer risk (under the additive genetic model, GG vs. GA vs AA, P = 0.042). Compared with genotypes containing the G allele (GG and GA), the AA genotype may decrease the risk of ovarian cancer (P = 0.0198, OR = 0.33, 95% CI [0.12∼0.78]). Compared with the G allele, the A allele is protective against ovarian cancer risk (P = 0.01756, OR = 0.66, 95% CI [0.46∼0.93]). Furthermore, a statistically significant association between the GG and GA+AA genotypes and the clinical stage was observed (P = 0.038). These data suggest that *MTDH* (−470G>A) could be a useful molecular marker for assessing ovarian cancer risk and for predicting ovarian cancer patient prognosis.

## Introduction

Ovarian cancer has the highest fatality rate of all female reproductive system malignancies, and in 2008 there were an estimated 225,500 new cases and 140,200 deaths worldwide [1]. As is the case for many malignancies, ovarian cancer is a multifactorial disease, and hormonal factors, wound healing and inflammation may all play a role in its development. Interactions between the environment and genetic factors also play significant roles [2]. Many studies have investigated the genetic basis of ovarian cancer susceptibility. For example, *BRCA1*, *BRCA2*, *MLH1*, *MSH2*, *RAD51C*, *RAD51D*, *RB1*, *SMAD6*, *CASP8*, and *LIN28B* have all been implicated in ovarian cancer [3], [4], [5], [6], [7], [8], [9], [10]. Recently, genome-wide association studies (GWAS) have found strong associations between ovarian cancer and several common susceptibility alleles in four loci [11], [12], [13]. Braem et al. reviewed 147 candidate genes, and the 3 GWAS studies published from 1990 to October 2010 identified approximately 1100 genetic variants in more than 200 candidate genes and 20 intergenic regions [8]. However, the relationships between known genetic variants and ovarian cancer are limited, and more studies need be performed to elucidate causal genetic variants and facilitate the identification of high risk subgroups within the general population [5].

*MTDH*, also known as astrocyte elevated gene-1 (*AEG-1*) and *Lyric*, was originally identified as an HIV-inducible gene in primary human fetal astrocytes [14]. *MTDH* is located at 8q22, consists of 12 exons and 11 introns and encodes a 582 amino acid protein with a calculated molecular mass of 64 kDa. *MTDH* over-expression has been detected in esophageal squamous cell carcinoma [15], gastric cancer [16], renal cancer [17], prostate cancer [18], non-small cell lung cancer [19], hepatocellular carcinoma [20], breast cancer [21]–[22], and neuroblastoma [23], compared to normal cells and the matched non-neoplastic regions [24]. The level of *MTDH* over-expression is significantly correlated with tumorigenesis, invasion, migration, progression, angiogenesis [25], EMT (epithelial mesenchymal transition), chemoresistance and radioresistance in various cancer types [17], [26], [27], [28], [29], [30], [31]. MTDH overe-xpression is correlated with peritoneal dissemination, lymph node metastasis, International Federation of Gynecology and Obstetrics stage, histological grade, presence of residual tumor and tumor recurrence in ovarian cancer [32], [33]. High *MTDH* expression was associated with the progression and prognosis of ovarian cancer [32], [33].

Our group has also previously found that variants in *MTDH* are significantly associated with breast cancer [34]. However, the association of *MTDH* variants with ovarian cancer susceptibility has not been investigated.

## Results

### The Relationship Between the *MTDH* (−470G>A) Polymorphism and Ovarian Cancer Risk

The participants in the case and control groups were all from mainland China. There were no significant clinical differences (i.e., body mass index [BMI], median age, menstrual history or other related parameters) between the 2 groups.

**Figure 1:**
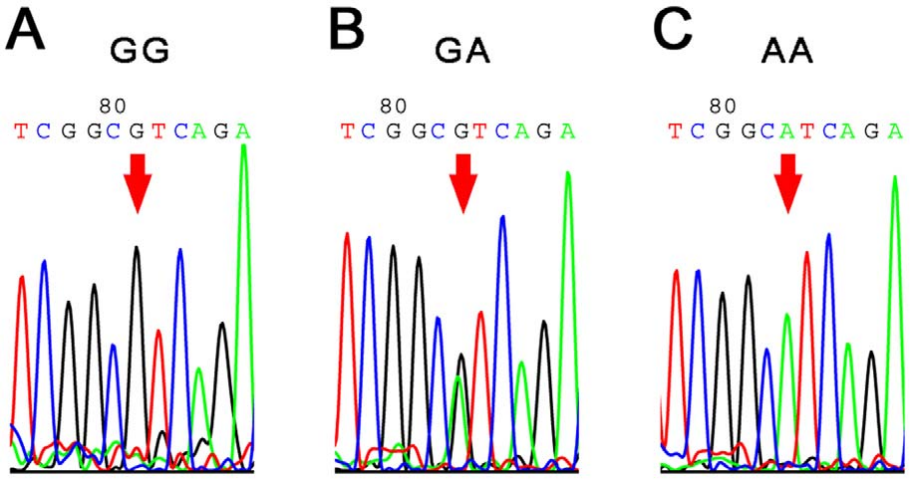
Sequencing chromatograms of *MTDH* (−470G>A). A–C, the sequencing chromatogram results of the genotype GG, GA and AA respectively. Samples were chosen randomly.

Hardy-Weinberg equilibrium showed that the chi-square values of the case group and control group were 0.1 and 3.94, respectively; both were p>0.05. As shown in Table 1, the *MTDH* (−470G>A) genotypes and allele distributions had a statistically significant difference between the case and control groups. We observed a statistically significant correlation with ovarian cancer risk (the additive genetic model, GG vs. GA vs AA, P = 0.042). Using the dominant genetic model (GG+GA vs AA), we observed a statistically significant difference in ovarian cancer risk (P = 0.0198, OR = 0.33, 95% CI [0.12 ∼0. 78]). These data showed that the homozygous AA genotype may be protective against ovarian cancer development and may decrease the risk of ovarian cancer. The A allele appears to be protective (P = 0. 01756, OR = 0.66, 95% CI [0.46∼0.93]) against ovarian cancer. Sequencing chromatograms from randomly chosen cases are used to illustrate the variants of *MTDH* (Fig. 1).

**Table 1:** The *MTDH* (−470G>A) genotype & Allele distribution in Ovarian Cancer and controls.

Genotype	Ovarian Cancer n(%)†	Controls n(%)†	P-value	OR 95%CI
GG	93(64.1)	141(55.5)	0.042	1.00 (reference)
GA	47(32.4)	88(34.6)		0.81 (0.52 ∼1.26)
AA	5(3.45)	25(9.84)		0.30 (0.11 ∼0.82)
GG+GA	140(96.6 )	229(90.2 )	0.0198	1.00 (reference)
AA	5(3.45 )	25(9.84 )		0.33 (0.12 ∼0.87)
GG	93(64.1 )	141(55.5 )	0.0924	1.00 (reference)
GA+AA	52(35.9 )	113(44.5 )		0.70 (0.46 ∼1.06)
G	233(80.3 )	370(72.8 )	0.01756	1.00 (reference)
A	57(19.7 )	138(27.2 )		0.66 (0.46 ∼0.93)

† The X^2^ for HWE of Ovarian Cancer group and control group is 0.1 and 3.94 respectively (both *P*>0.05).

### The Relationship between the *MTDH* (−470G>A) Polymorphism and Clinicopathological Variables

Table 2 shows the association of the GG and GA+AA genotypes with clinicopathological characteristics, including age at diagnosis, degree of tumor differentiation, clinical stage, lymph node metastasis, CA125 expression, tumor size and tumor histology. A statistically significant association with clinical stage was found (P = 0.038). No relationships between the polymorphism and age at diagnosis, degree of tumor differentiation, lymph node metastasis, CA125 expression, tumor size or tumor histology were found. Thus, the *MTDH* (−470G>A) polymorphism may be an indicator of clinical stage in ovarian cancer.

**Table 2:** Results of association analysis between rs16896059 and clinicopathological characteristics.

Clinical data information	All(%)	Genotype		P-value	OR
		GG(%)	GA+AA(%)		
Age					
≤50	43(31.9)	31(23.0)	12(8.89)	0.608	1.00(reference)
>50	92(68.1)	61(45.2)	31(23.0)		1.206
Degree of Differentiation					
Low	89(84.0)	58(54.7)	31(29.2)	0.617	1.00(reference)
Middle & High	17(16.0)	10(9.43)	7(6.60)		0.764
Clinical stage					
I & II	36(28.8)	18(14.4)	18(14.4)	0.038	1.00(reference)
III & IV	89(71.2)	62(49.6)	27(21.6)		2.296
Positive lymph node					
Negative	37(66.1)	20(35.7)	17(30.4)	0.515	1.00(reference)
Positive	19(33.9)	12(21.4)	7(12.5)		1.457
CA125					
≤65(U/ml)	23(17.6)	15(11.5)	8(6.11)	0.962	1.00(reference)
>65(U/ml)	108(82.4)	71(54.2)	37(28.2)		1.023
Size of tumor					
<10 cm	80(59.3)	54(40.0)	26(19.3)	0.496	1.00(reference)
≥10 cm	55(40.7)	34(25.2)	21(15.6)		0.780
Tumor histology					
Serous	89(65.4)	58(42.6)	31(22.8)	0.877	1.00(reference)
Other	47(34.6)	30(22.1)	17(12.5)		0.943

### The Relationship Between the *MTDH* (−470G>A) Polymorphism and MTDH Protein Levels

**Figure 2:**
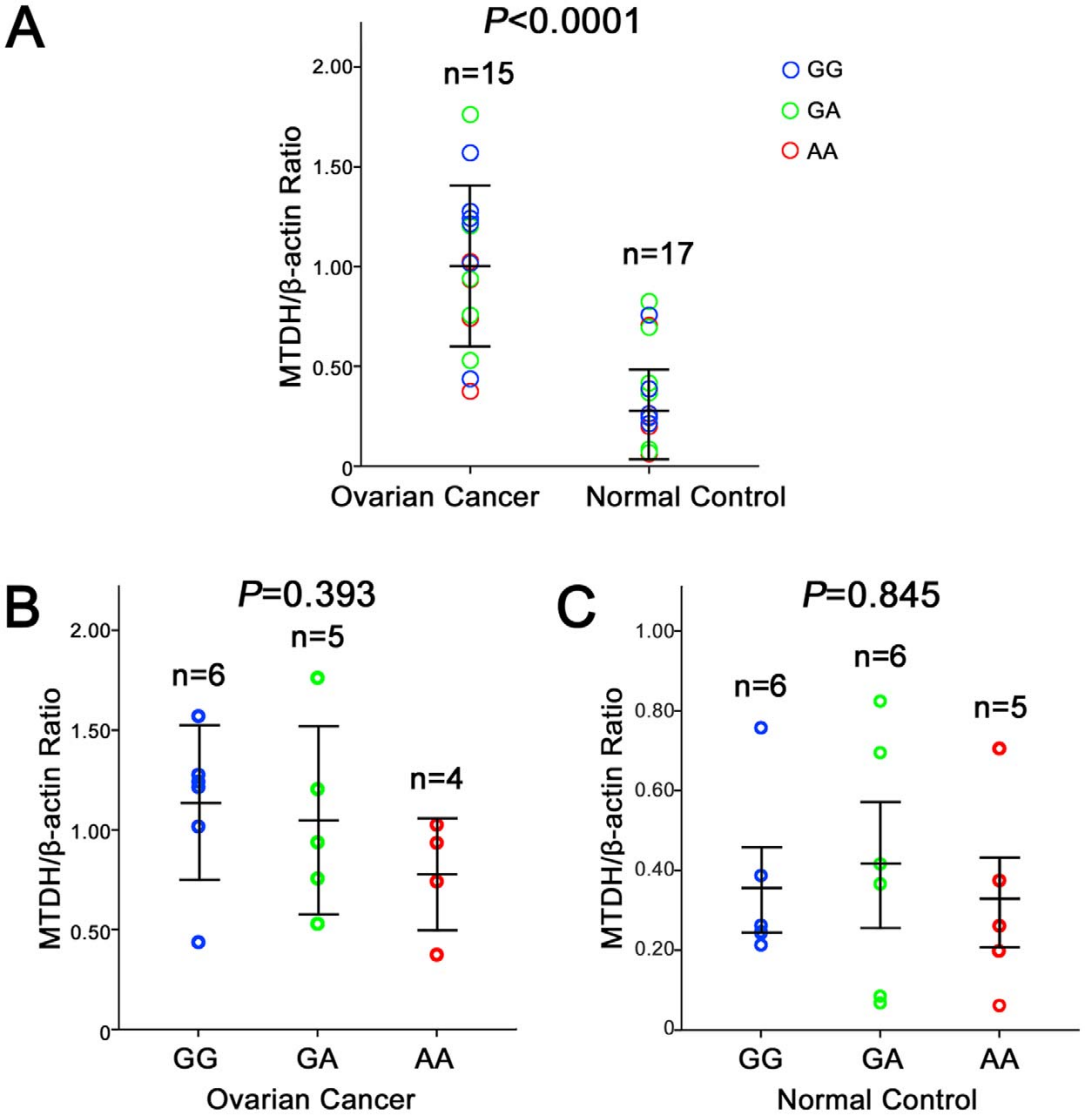
Association of the −470G>A genotype and *MTDH* (−470G>A) protein expression. A, Relative level of MTDH protein expression in ovarian cancer tissues compared to normal ovarian tissues. B, Relative level of MTDH protein expression in the ovarian cancer tissues of patients with different −470G>A genotypes. C, Relative level of MTDH protein expression in normal tissues of individuals with different −470G>A genotypes. One circle represents the mean of three independent measurements from one patient. The distribution of the three genotypes were random between the groups. N represents the samples number of respective group. Bars represent the standard deviation. Student’s t test was used to evaluate the differences in the expression levels of different constructs.

We further studied the possible relationship between the *MTDH* (−470G>A) polymorphism and the MTDH protein expression level in vivo. As shown in Fig. 2, the levels of MTDH protein in 15 ovarian cancer tissues were significantly higher than those in 17 normal tissues (P<0.01). As shown in Fig. 2B and 2C, the MTDH protein expression was higher in patients with the GG or GA genotypes than in those with the AA genotype, but these differences were not significant (P>0.05). These findings suggest that the SNP −470G>A may not significantly impact the expression of the *MTDH* gene.

## Discussion

*MTDH* plays a critical role in tumor biology, and it is involved in a variety of tumor biological behaviors. Our group was the first to discover a significant association between *MTDH* and tumor susceptibility: that is, that 2 *MTDH* variants are associated with breast cancer [34]. In this study, we further analyzed various SNPs in *MTDH* and found another SNP (rs16896059, −470G>A) with statistically significant differences between ovarian cancer case and control groups. Furthermore, rs16896059 was statistically significantly associated with the clinical stage of the tumor. To the best of our knowledge, this is the first variant association study of *MTDH* SNPs and ovarian cancer risk. We expect that *MTDH* will be a useful molecular marker for assessing ovarian cancer risk and for predicting ovarian cancer patient prognosis. However, this finding should be verified in a larger sample.

The SNP rs16896059 is located in the promoter of *MTDH* and thus polymorphisms do not alter the sequence or structure of the protein. We investigated the protein expression level of MTDH by western blotting. As previously reported, the expression levels of MTDH in ovarian cancer tissues were significantly higher than the levels in adjacent normal tissues (P<0.0001) [32], [33]. However, we did not find any statistically significant effect of the −470G>A SNP on the protein expression of the *MTDH* gene in ovarian cancer tissues or the normal tissues. Thus, no impact of the SNP on *MTDH* expression was evident. Because of the −470G>A SNP was located in the promoter region, and then it could also affect promoter activeity. Therefore, the association of the *MTDH* (−470G>A) polymorphism with *MTDH* promoter activeity and its effect on ovarian cancer development should be studied in vitro to further investigate the molecular mechanisms involved.

As indicated above, most patients who participated in our study were living in Shandong Province, China. Due to the general genetic homogeneity of this ethnic population, we speculate that these findings will be consistent in larger sample sizes across China. However, the relationship between *MTDH* polymorphism and ovarian cancer risk requires further investigation in different ethnic populations [34].

In conclusion, the A allele of the *MTDH* SNP rs16896059 (−470G>A) is protective against ovarian cancer, and the homozygous AA genotype may be a protective genotype. The polymorphism is statistically significantly associated with clinical stage.

## Materials and Methods

### Patients and Samples

The study was approved by the Ethical Committee of Shandong University. All participants gave written informed consent to participate in this study. 145 patients (mean age of 51.8±13.1 years) participated in the study, diagnosed with ovarian cancer in Qilu Hospital of Shandong University between September 2008 and July 2011. Clinical data information, including age at diagnosis, degree of differentiation, clinical stage, positive lymph node, CA125, size of tumor and tumor histology were obtained from patients’ medical records. 254 age-matched healthy women (mean age of 49.2±12.8 years) were recruited as control. Most participants were Han Chinese residing in Shandong Province, China. DNA from peripheral blood cells s was extracted with TIANamp Genomic DNA Kit (Tiangen, Beijing, China), by instructions. The DNA purity and concentration were measured by ultraviolet spectrophotometer (GE Healthcare, USA). DNA samples were conventionally stored at −80°C as previously described [34], [35].

### Genotyping Analysis of the *MTDH* (−470G>A)

Genotyping of the SNP rs16896059 (−470G>A) polymorphism was determined by PCR and sequencing method. The sequence of *MTDH* gene was obtained from NCBI (Gene ID: 92140, Nucleotide: AC_000140.1, GI: 157734173). Primers were designed with Primer Premier 5 according to the sequence of rs16896059 as follows: forward primer 5′- CTGGCAACTGGTAGGCACGC -3′ and reverse primer 5′- GAGGGACTCGCAGGATGACG -3′. The PCR productions size was 893 bp. PCR amplification was performed in 50 µl reaction systems containing 1 µl genomic DNA (100 ng/µl), 4 µl of 2.5 mM dNTPs, 10 µl buffer, 2 µl of each primer(0.4 µM) and 0.5 U PrimeSTAR HS DNA Polymerase (TAKARA, DALIAN, China). The PCR amplification conditions were: 94°C for 5 min, 35 cycles at 98°C for 10 seconds, 55°C for 15 seconds, and 72°C for 2 min, and a final extension step of 72°C for 5 mins. All PCR productions were sequenced by BioSune Biotechnology Co., Ltd. (Shanghai, China), and the sequence data were analyzed with Chromas 2.31 and MegAlign 7.0 software.

### Western Blot

Western blot analysis was performed as previously described [31], [36], Tissue was dissolved in RIPA buffer (1×PBS, 0.1% sodium dodecyl sulfate, 1% NP40, 5 mM EDTA, 1 mM sodium orthovanadate, 0.5% sodium deoxycholate and protease inhibitors). 40 µg of proteins were electrophoresed on 10% SDS-PAGE and transferred onto PVDF (Millipore) using a Mini Trans-Blot Cell (Bio-Rad Laboratories, Hercules, CA, USA). Immunoblotting was carried out using a primary antibody *MTDH* (ab45338, 1∶500, Abcam, Cambridge, MA, USA) and β-actin (1∶5,000, Sigma-Aldrich, St. Louis, MO, USA). The secondary antibodies (1∶5,000, KPL, Gaithersburg, MD, USA) were labeled by HRP (horseradish peroxidase). Membranes are visualized using an ECL kit (Merck, Darmstadt, Germany). The loading control was β-actin.

### Statistical Analysis

The statistical data were analyzed as previously described [34]. Hardy-Weinberg equilibrium test was performed d using a web-based statistical tool OEGE (http://www.oege.org/software/hwe-mr-calc.shtml). The genotype and allele distributions in the ovarian cancer groups and control groups were analyzed using chi-squared tests, and Fisher’s exact test was used when one cell count was <5. The risk of ovarian cancer development was estimated as an odds ratio (OR) with a 95% confidence interval (CI) using an unconditional logistic regression analysis. All statistical tests were two-sided with a significance level of p≤0.05. The statistical data were analyzed with SPSS 17.0 (SPSS, Chicago, Illinois, USA).
